# Prediction of twin-arginine signal peptides

**DOI:** 10.1186/1471-2105-6-167

**Published:** 2005-07-02

**Authors:** Jannick Dyrløv Bendtsen, Henrik Nielsen, David Widdick, Tracy Palmer, Søren Brunak

**Affiliations:** 1 Center for Biological Sequence Analysis BioCentrum-DTU Building 208 Technical University of Denmark DK-2800 Lyngby, Denmark; 2 Department of Molecular Microbiology John Innes Centre Norwich NR4 7UH UK; 3 School of Biological Sciences University of East Anglia Norwich NR4 7TJ UK

## Abstract

**Background:**

Proteins carrying twin-arginine (Tat) signal peptides are exported into the periplasmic compartment or extracellular environment independently of the classical Sec-dependent translocation pathway. To complement other methods for classical signal peptide prediction we here present a publicly available method, TatP, for prediction of bacterial Tat signal peptides.

**Results:**

We have retrieved sequence data for Tat substrates in order to train a computational method for discrimination of Sec and Tat signal peptides. The TatP method is able to positively classify 91% of 35 known Tat signal peptides and 84% of the annotated cleavage sites of these Tat signal peptides were correctly predicted. This method generates far less false positive predictions on various datasets than using simple pattern matching. Moreover, on the same datasets TatP generates less false positive predictions than a complementary rule based prediction method.

**Conclusion:**

The method developed here is able to discriminate Tat signal peptides from cytoplasmic proteins carrying a similar motif, as well as from Sec signal peptides, with high accuracy. The method allows filtering of input sequences based on Perl syntax regular expressions, whereas hydrophobicity discrimination of Tat- and Sec-signal peptides is carried out by an artificial neural network. A potential cleavage site of the predicted Tat signal peptide is also reported. The TatP prediction server is available as a public web server at .

## Background

The Tat (Twin arginine translocation) pathway, which operates in parallel to the well characterized Sec export pathway, has recently been discovered in bacteria [1-3]. Substrates of the Tat pathway are often redox cofactor binding proteins which acquire their cofactors, and therefore fold in the cytoplasm. Thus, in contrast to protein export via the Sec pathway, Tat substrates are folded prior to export [1,4]. Indeed, there is good evidence to suggest that the Tat pathway has the ability to recognize the folded state of a substrate protein and to reject unfolded proteins [5,6].

**Figure 1:**
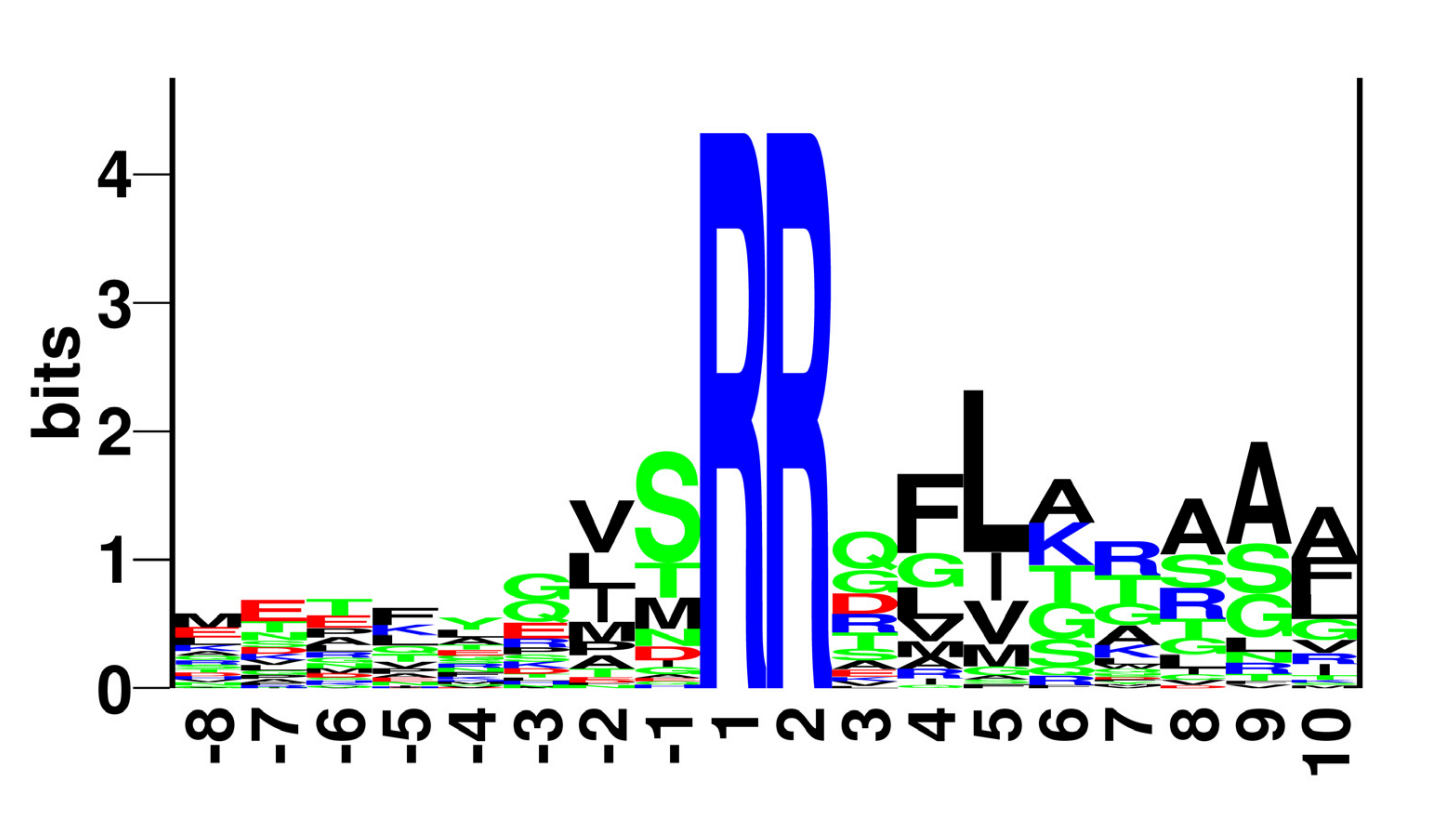
**Tat signal peptides. **Sequence logo of the positive training set aligned at the two consecutive arginines. None of the variant forms of Tat signal peptides were included. Sequence conservation is shown in bits and constructed as previously described [29].

Proteins entering the Tat pathway have signal peptides with a tripartite structure that is much like classical Sec signal peptides and indeed are probably also cleaved by leader peptidase [7]. However, in contrast to Sec signal peptides, a striking twin-arginine motif is found at the border between the n- and h-regions of the Tat signal peptide. A consensus sequence for this twin-arginine motif has previously been defined as (S/T)RRxFLK [1], where the two consecutive arginines are invariant (see Figure 1). With an average length of 37 amino acids, Tat signal peptides are significantly longer than classical Sec signal peptides. (The extended length is usually found in the n-region). In addition, the h-region of Tat signal peptides has a lower average hydrophobicity than classical Sec signal peptides [8]. Tat signal peptides are not limited to the bacterial domain, and they have also been found in Archaea [9] and in plants where they mediate Tat-dependent translocation across the chloroplast thylakoid membrane [10].

Although the twin-arginine sequence motif has previously been defined, some variations of this may still be accepted by the Tat machinery. For example, the tetrathionate reductase from *Salmonella enterica *was found to lack one of the (previously thought) invariant arginines of the twin-arginine motif [11]. Moreover, analysis of the *E. coli *pre-propenicillin amidase Tat signal peptide showed that it targets the Tat pathway. This signal peptide deviates from the consensus by having an asparagine between the two arginines in the twin-arginine motif [12]. On several occasions, mutational analysis has shown that the two consecutive arginines are not absolutely required for export via the Tat translocon. Either arginine of the *E. coli *SufI protein could be substituted by lysine without blocking Tat-dependent transport, although the rate of transport was reduced [13]. Substituting both arginines for lysine, or the first arginine for alanine, blocked transport completely. A similar result was found by Ize *et al. *[14], working with the *E. coli *TorA Tat signal peptide fused to green fluorescent protein (GFP) [14]. Buchanan *et al. *[15] found that the first arginine of the *E. coli *TorA Tat signal peptide could be substituted for lysine without blocking Tat-dependent transport [15]. Also using a TorA-GFP construct, DeLisa *et al. *[16] found that the second arginine could be substituted not only by lysine but also by asparagine or glutamine without blocking export.

Numerous studies have used regular expressions in combination with SignalP for identification of putative Tat signal peptides. The SignalP method for prediction of Sec signal peptides [17,18], can to some extend correctly predict Tat signal peptides; although the h-regions of Tat signal peptides tend to be less hydrophobic than classical Sec signal peptides and SRP signal sequences. However, accuracy by the SignalP approach for prediction of Tat signal peptides is low, and the position of the predicted cleavage site is often wrongly assigned. This is probably due to the extended length of the Tat signal peptide and the fact that frequently the c-regions of Tat signal peptides contain basic amino acids [8].

Two different targeting pathways converge at the Sec translocon. Proteins carrying SRP (signal recognition particle) signal sequences are targeted via SRP to the Sec machinery, mostly for integration into the membrane. Proteins carrying Sec signal peptides are translocated across the bacterial membrane and the signal peptide is cleaved off in order to release the mature protein. Discrimination of Sec and SRP signals is apparently based on the hydrophobicity of the signal sequence itself. Increasing the hydrophobicity of a Sec signal peptide reroutes it to the SRP targeting pathway [19,20].

Recently, a Perl program for prediction of Tat signal peptides, TATFIND, based on regular expressions was developed [9,21]. In addition to regular expressions, TATFIND uses a hydrophobicity measure in a rule based classification scheme. Because of a limited number of variant Tat signal peptides (not containing two consecutive arginines), the program was designed to ignore these. Therefore it is not capable of identifying the variant Tat signal peptides unless the user possesses some programming experience for changing the TATFIND program code.

In this report we describe a new method, termed TatP, for the identification of Tat signal peptides. TatP differs from the previously described TATFIND because it integrates pattern matching and machine learning. Comparison of TatP with TATFIND on various independent test sets indicates that TatP has slightly more false negative predictions than TATFIND. Nevertheless, TatP generates far less false positive predictions than TATFIND.

## Implementation

### Data set extraction

All sequence data were extracted from Swiss-Prot release 42.0 [22]. We extracted putative Tat signal peptide sequences with the database cross-reference identifier (DR: TAT_signal_seq) from the TIGR database of protein families (Ac: TIGR01409). The TIGR family of Tat signal peptides is based on predictions using a hidden Markov model.

Of the 117 sequences extracted matching the above criteria, twelve were found by comments in the 'CC -!- PTM' line to have experimental evidence for utilizing the Tat export pathway and were removed from the positive training set into an independent test set (see below). Due to limited amount of data, the positive training set was homology partitioned, thereby bringing the most homologous sequences together into 5 different subsets .

1178 cytoplasmic sequences all carrying two consecutive arginines (RR) within the first 30 amino acids were extracted from Swiss-Prot. Following redundancy reduction, a resulting set of 462 cytoplasmic sequences together with 62 Gram-positive and 70 Gram-negative Sec signal peptides randomly chosen from the SignalP 3.0 dataset without the 'Tat_signal_seq' identifier, were used as a negative training set. The negative training set was homology reduced using the same scheme as for SignalP [17,23]. In the negative training set we do not distinguish between Sec signal peptides or SRP signal sequences.

Twelve Tat substrates (DMSA_ECOLI, MBHS_ALCEU, MBHS_ALCHY, MBHS_AZOVI, MBHS_ECOLI, MBHS_OLICA, MBHS_WOLSU, MBHT_ECOLI, OPGD_ECOLI, PHNS_DESVM, PHSS_DESBA, TORA_ECOLI) were removed from the positive training set into an independent test set, together with two additional sequences from a mutation study of TorA [8]. This test set of known Tat substrates is biased, as most of these proteins function as the small subunit of [Ni-Fe] hydrogenases. Therefore, we also included additional 23 Tat substrates from *E. coli *in the independent test set, having a total of 35 known Tat substrates. A curated set of *E. coli *Tat signal peptides can be found at .

Furthermore, a test set of 15 sequences all with putative Tat signal peptides from *B. subtilis*, was found by literature studies [24,25].

Additionally, we have used the remaining 713 bacterial cytoplasmic sequences (removed by homology reduction) all carrying two consecutive arginines (RR) within the first 30 amino acids of each sequence as an evaluation set, as well as a test set of 632 transmembrane proteins also carrying two consecutive arginines within the initial 30 amino acids.

### Regular expressions

Perl syntax regular expressions are allowed as filtering of potential Tat signal peptides. Furthermore, this assists the user in identifying the position of a potential Tat signal peptide motif. A regular expression of 'RR. [FGAVML] [LITMVF]', where '.' means any amino acid, is used by default and this regular expression covers 97% of the sequences found in the training set. This pattern was extracted from an ungapped multiple sequence alignment of the positive training set where the position of the two arginines was fixed.

### Neural network architecture

We used two different neural networks for coping with the Tat signal peptide prediction problem. One network for recognition of the cleavage site, and one network for determining whether a given amino acid belongs to the Tat signal peptide or not (discrimination). A more thorough description of the neural network has been presented in previous papers [17,23].

Performance evaluation of the prediction method was carried out on different data sets. During training of the method, we used the same measure of performance as used in SignalP *i.e.*cross-validation on the training set. This means that the data set was split into five equally sized parts followed by training on four and tested on one for a total of five times until all sequences independently had been used for training and testing, respectively.

Furthermore, we evaluated the method on various independent test sets as described above.

The cleavage site network was based on an asymmetric window of 19 positions to the left and 3 positions to the right of the cleavage site. The cleavage site network had 2 units in the hidden layer. The discrimination network performs best using a symmetric or nearly symmetric window. The window size used in this work was symmetric covering 31 positions. The discrimination network had 3 units in the hidden layer. Composition and positioning information was included in the neural network as previously described [17].

### Description of scores

Five different scores are presented in the output of TatP. Two scores, the S-score and the C-score, are generated by the discrimination and cleavage site network and are calculated for each position in the amino acid sequence. The positioning of the predicted cleavage site is indicated by the Y-score. The Y-score is defined as,



where *Δ*_*d*_*S*_*i *_is the difference between the average S-score of d positions before and d positions after position i:



The mean S-score is calculated as the average of the S-score in the predicted signal peptide region separated by the Y-score for maximal cleavage site prediction.

As for SignalP version 3.0, the best discrimination of signal peptides versus non-signal peptides, is calculated as an average of the mean S-score and the maximal Y-score. We call it the D-score. If the D-score is above the defined cutoff value and a Tat signal peptide motif is found the sequence is nominated as a Tat substrate.

## Results and Discussion

### Performance evaluation of TatP

The TatP prediction server is a regular expression and neural network based tool that can be used to predict the presence of bacterial Tat signal peptides. The user is allowed to independently specify a preferred consensus pattern by Perl regular expressions as a filtering of input sequences. As default, we have implemented a complex consensus pattern which covers 97% of putative Tat signal peptides in the training set. The used regular expression is generated from the training data and can be identified in 103 of the 105 positive training sequences. The reason for excluding two sequences was to generate a consensus pattern that was as strict as possible. We allow the user to change the consensus pattern before running the entire prediction, which allows for large flexibility of the method. In eleven of the 105 positive training sequences, TATFIND did not identify a Tat signal peptide motif. The strength of allowing the user to choose a preferred consensus pattern is obvious when it comes to prediction of variant Tat signal peptides, which under default conditions are discarded. Our method uses a regular expression for identifying putative Tat substrates and a neural network for coping with the less hydrophobic h-region of the Tat signal peptide. The h-region is generally less hydrophobic in Tat signal peptides than classical Sec or SRP targeting sequences.

**Figure 2:**
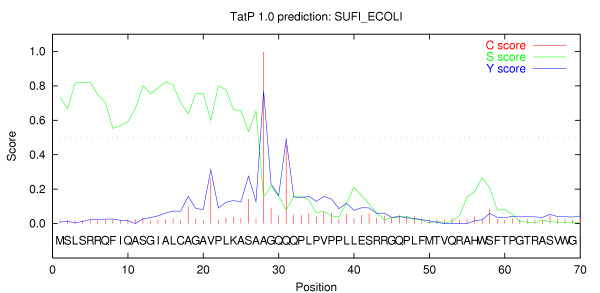
**Server output. **Graphical output from the prediction server. Here is shown the prediction results from SUFI_ECOLI, a known Tat substrate. The S-score indicates whether an amino acid belongs to a Tat signal peptide or not. C- and Y-scores indicate the positioning of a potential cleavage site.

A numeric and graphic output is generated to assist the user to interpret borderline cases and localize the predicted Tat signal peptide cleavage site (See Figure 2).

For evaluation of TatP, we have tested the performance of our method in several different ways. We have evaluated the method on twelve experimentally verified Tat signal peptides extracted from Swiss-Prot together with a curated set of *E. coli *Tat signal peptides. Furthermore, we have analyzed sequences of TorA (*E. coli *TMAO reductase) where the wildtype signal peptide targets a passenger protein to the Tat pathway, and where mutational analysis shifted the targeting of the protein to the Sec pathway. We also examined whether the method is able to discriminate the Sec signal peptides emerging from the generation of the training set for SignalP version 3.0 from Tat signal peptides [17]. A proteomic study on proteins secreted in *Bacillus subtilis *was also used for validation of the TatP method [24,25]. Moreover, we also comment on a few known cases of the variant Tat signal peptides, as well as prediction on transmembrane helices.

### Discrimination between Sec and Tat signal peptides

Numerous studies have used SignalP for prediction of Tat signal peptides. To some extent, SignalP is able to correctly classify Tat signal peptides, although the accuracy is lower than the TatP method presented here.

Of the 35 known Tat substrates used in this study, only 83% receive a positive prediction by SignalP, even though they all have the consensus pattern. This shows that the SignalP can be used for Tat signal peptide prediction, although a more accurate method is preferable. The method presented here – TatP – has a higher accuracy than the approach mentioned above. The TatP method predicts 91% of the 35 known Tat substrates as having a Tat signal peptide. This is comparable to TATFIND which positively predicted 97% of these. Unfortunately, three known Tat substrates were not correctly identified by TatP, PHSS_DESBA, YAGT_ECOLI and FHUD_ECOLI. TATFIND was not able to correctly classify YCDO_ECOLI where it found no consensus motif for a Tat signal peptide.

In addition to the classification prediction, TatP also reports a potential cleavage site for the Tat signal peptide (see Figure 2), a feature which is not reported in the output by TATFIND. Unfortunately, not all of the 35 known Tat signal peptides have experimentally verified cleavage sites. Merging information from Swiss-Prot and our curated *E. coli *dataset we expect to have a correct cleavage site annotation in all known Tat substrates. When using the merged annotations, the TatP method has a correct cleavage site prediction in 84% of true-positive predicted Tat substrates.

Assessment of whether TatP and TATFIND were able to discriminate Tat signal peptides from Sec signal peptides was carried out on the training set for SignalP version 3.0. Additionally, we evaluated the performance of TatP and TATFIND on 713 cytoplasmic proteins carrying two consecutive arginines located within the initial 30 amino acids. By searching these datasets with the regular expression pattern alone, we find that the false positive rates are 4% and 7% for Gram-positive and Gram-negative proteins, respectively. For cytoplasmic proteins with two consecutive arginines within the initial 30 amino acids, the false positive rate of the regular expression alone is 15%. TATFIND greatly reduces the amount of false positive predictions by including a rule-based classification scheme in addition to regular expressions, resulting in a false positive rate of 1.4%. In TatP we apply a neural network to identify the less hydrophobic stretches of the h-region in Tat signal peptides and the false positive rate is lowered to only 0.4% (see Table 1). Indeed, this shows the power of the neural network, which is capable of discriminating between the hydrophobicity of Sec/SRP and Tat targeting signals. In the Gram-positive test set, only slightly more false positives were found by TatP than by TATFIND.

**Table 1:** False positive predictions. The table shows the rate of false positive predictions on Gram-positive, and Gram-negative proteins which are secreted via the Sec-dependent pathway. Furthermore is shows cytoplasmic proteins and transmembrane helix proteins, both carrying two consecutive arginines within the initial 30 amino acids. Used methods for prediction are regular expression (Regex), TATFIND and TatP (regular expressions in combination with a neural network (NN)).

**Method**	**Gram+**	**Gram-**	**Cyt. (RR)**	**Transmem (RR)**
Regex	4%	7%	15%	23%
TATFIND	1%	6%	1.4%	7.0%
TatP (Regex+NN)	3%	5%	0.4%	3.5%

It should be mentioned that some of these false positive predictions by TatP and TATFIND might represent true Tat signal peptides in the database. The used Gram-negative and Gram-positive data sets were extracted from Swiss-Prot before Tat signal peptides were annotated as such. Moreover, many of these sequence entries were entered in the Swiss-Prot database even before the Tat export pathway was discovered, thus a few Tat signal peptides might be erroneously annotated.

Positive amino acid residues have a topological effect on transmembrane helices [26]. It is interesting to see whether TatP and TATFIND are able to discriminate transmembrane helices from Tat signal peptides. 632 proteins carrying transmembrane domains and also carrying two consecutive arginines within the initial 30 amino acids were analyzed. We found 22 proteins to be predicted as having Tat signal peptides, whereas TATFIND identified 46. Many of these were indeed metalloproteins but had a transmembrane domain annotation instead of a Tat signal peptide annotation. The reasons for this may be the same as described above. Rieske proteins remain anchored to the membrane by an uncleaved Tat signal sequence in thylakoids [27], which is also believed to be the case in prokaryotes since the Fe-S cluster of the protein must reach the trans-side of the membrane. The exact amount of true Tat signal peptides in this transmembrane data set is unknown. Nevertheless, it shows the power of TatP to identify putative Tat-substrates which are erroneously annotated as being transmembrane helices. TATFIND seems to over-predict the transmembrane proteins having two consecutive arginines, as some of the 46 predicted proteins are chaperones, which are unlikely to carry a Tat signal peptide. Prompted by the lack of experimental evidence for a large number of Tat substrates, we looked at 22 bacterial genomes, where genes encoding components of the Tat translocation system failed to be identified on the basis of homology [21]. Ideally, no Tat substrates should be found in these genomes by any Tat signal peptide prediction method. In these 22 bacterial genomes, TATFIND identified 20 Tat substrates whereas TatP identified only 4.

Cristóbal *et al. *[8] showed that Tat signal peptides are generally less hydrophobic than both Sec signal peptides and SRP signal sequences. They demonstrated that the Tat signal peptide of the *E. coli *TMAO reductase (TorA) could be converted into a Sec signal peptide without changing the RR motif, but instead replacing by the hydrophobic h-region with a more hydrophobic region taken from a Sec signal peptide. Both TorA signal peptide mutants and the wildtype Tat signal peptide carry the consensus pattern. According to the hydrophobicity discrimination from the neural network, the wildtype TorA is correctly predicted to be secreted via the Tat pathway. The TorA [10:10] signal peptide mutant, which is more hydrophobic than the wildtype but having the same length, is falsely predicted positive, while the TorA [19:10] signal peptide mutant, which is both more hydrophobic and shorter, is correctly predicted negative. Similar results are found for TATFIND.

### Tat signal peptides in B. subtilis

Jongbloed et al. [24] compiled a list of 69 *B. subtilis *proteins with potential Tat signal peptides. In the light of the above-mentioned mutation studies which showed that the first arginine in some cases could be substituted by a lysine without loss of Tat-dependence, they considered both RR- and KR-motifs. All sequences with an RRXΦΦ or KRXΦΦ (where Φ denotes a hydrophobic residue) followed by a hydrophobic region were selected from the genome sequence. Subsequently, the authors looked for the selected proteins in the *B. subtilis *secretome. Strikingly, only one of the 69 proteins was actually found to be Tat-dependent, the phosphodiesterase PhoD. It is positively predicted by TatP. 13 other proteins were found to be secreted, but were also secreted by a *tat *deletion mutant, indicating Sec-dependence. All of these 13 proteins carry a sequence matching the consensus motif, but after applying the neural network for analyzing the hydrophobicity of these signal peptides all 13 receive a negative and correct prediction by TatP. Three of the 13 proteins, LipA, WapA and YolA, were furthermore shown to be dependent on SecA [24]. TATFIND made one false positive prediction, but was also capable of correctly predicting twelve Sec-dependent proteins not to carry a Tat signal peptide. The above mentioned sequences were all predicted to have a Sec signal peptide using SignalP version 3.0 (without truncation), except for PhoD (See Table 2). Thus, in the case of *B. subtilis*, putative Tat signal peptides were all correctly classified as being Sec signal peptides by SignalP and none of them received a positive prediction by TatP, showing the power of regular expression based searches also incorporating the neural network for hydrophobicity discrimination.

**Table 2:** Putative Tat substrates in *B. subtilis*. The table shows the predictions by SignalP version 3.0, regular expressions, TATFIND and TatP on *B. subtilis *proteins which have a putative Tat signal peptide motif, but nevertheless are secreted via the Sec-dependent pathway. Only PhoD and YwbN are secreted via the Tat secretion pathway.

**Protein**	**Regex**	**SignalP**	**TATFIND**	**TatP**
LipA	Yes	Yes	No	No
AbnA	Yes	Yes	No	No
BglC	Yes	Yes	No	No
BglS	Yes	Yes	No	No
LytD	Yes	Yes	No	No
OppA	Yes	Yes	No	No
PbpX	Yes	Yes	No	No
WapA	Yes	Yes	No	No
WprA	Yes	Yes	No	No
YdhF	Yes	Yes	No	No
YfkN	Yes	Yes	Yes	No
YhcR	Yes	Yes	No	No
YolA	Yes	Yes	No	No
PhoD	Yes	No	Yes	Yes
YwbN	Yes	No	Yes	Yes

Very recently, a second Tat-dependent substrate was identified in *B. subtilis*. YwbN was found to be secreted in a strictly Tat-dependent manner [25]. YwbN is positively and correctly predicted by TatP.

In the *B. subtilis *genome, TATFIND predicts seven Tat signal peptides [21], while TatP with the default regular expression predicts five. If we follow Jongbloed et al.'s idea and allow KR- as well as RR-motifs (but keep the rest of the regular expression unchanged), the number of positive predictions is ten. The exact number of Tat substrates in this organism, however, must await experimental verification.

### Variant Tat signal peptides

TatP without the regular expression (or with a variant regular expression) could potentially predict variant Tat signal peptides without the canonical twin arginines. The TtrB subunit of *Salmonella enterica *tetrathionate reductase carrying a lysine instead of an arginine in the first position of the Tat signal peptide motif (Genbank AC: NP_805056) [11], was correctly and positively predicted by TatP if the regular expression was expanded with a lysine in the first position. TATFIND does not directly allow variations in the used regular expression (consensus motif), although it can be changed if one has basic programming skills. Thus, the TtrB subunit receives a negative prediction by TATFIND. Moreover, the mutant Tat signal peptides generated by Stanley *et al. *[13], Buchanan *et al. *[15], Ize *et al. *[14], and DeLisa *et al. *[16] are easily predicted by TatP if the user modifies the regular expression to allow for the relevant substitutions in the RR positions. However, the naturally occurring variant Tat signal peptide reported by Ignatova *et al. *[12] was unfortunately not predicted positively by TatP.

We did not specifically investigate Tat signal peptides fused to heterologous passenger proteins [5,28]. However, we expect the majority of these fusion proteins to obtain a correct prediction (with the caveat that the passenger protein is competent for export by the Tat pathway) as the prediction method mainly uses the N-terminal region of the protein sequence.

### Database annotations

As described above, errors can enter a database due to lack of knowledge. Many Tat signal peptides are annotated as Sec signal peptides, simply because the corresponding sequences entered the database before any knowledge of the Tat secretion pathway.

From release 42.0 to 44.0 of Swiss-Prot many new Tat signal peptides have been annotated using the TIGR HMM (see Methods and Materials). Even though Swiss-Prot is manually curated, errors have been found regarding annotation of Tat signal peptides. It is doubtful whether the following Swiss-Prot entries carry Tat signal peptides, as the Tat secretion pathway so far has not been identified in eukaryotic ER membrane translocation. We have found the following six eukaryotic sequences carrying the TIGR Tat signal peptide identifier (TAT_signal_seq), which did not have associations with chloroplast thylakoid membrane trafficking, CLC7_HUMAN, CLC7_MOUSE, CLC7_RAT, CLCC_ARATH, CO1B_HUMAN and PSP1_ARATH. The above mentioned cases seem to be false positive predictions made by the TIGR HMM.

## Conclusion

The TatP method presented here integrates regular expressions and a neural network based method for prediction of twin-arginine (Tat) signal peptides. Our study shows that regular expressions alone are not capable of correctly identifying Tat signal peptides. The further addition of a neural network aids correct classification of Tat signal peptides and Sec signal peptides. Although the TatP method only slightly outperforms TATFIND, the method presented here is far superior to a combination of simple pattern matching and classical signal peptide prediction methods. Thus, this method is highly useful as it is publicly available as an easy to use online prediction server. In the area of investigating variant Tat signal peptides, no programming skills are needed.

We believe that the TatP prediction method will serve as a nice complement to the SignalP method for prediction of both Tat and classical Sec signal peptides.

## Availability and requirements

The TatP prediction server is available at  and the SignalP server for prediction of classical Sec signal peptides is available at . The TatP prediction method is available for the IRIX, SunOS and Linux platforms and is dependent on a range of standard Unix tools. A license for both academic and non-academic users can be obtained by following the instructions found on the above web page.

## Authors' contributions

JDB carried out sequence retrieval, neural network training and optimization and drafted the manuscript. HN provided input on the manuscript and method. DW tested the robustness of the method. TP provided a curated *E. coli *dataset and wrote parts of the biological background. Finally, SB provided general inputs and improvements to the manuscript.
